# Mycobacterium tuberculosis growth inhibition by peripheral blood mononuclear cells from household contacts is not affected by previous SARS-CoV-2 infection

**DOI:** 10.12688/gatesopenres.16362.1

**Published:** 2025-09-09

**Authors:** Jane A Shaw, Caleb Petersen, Andriette Hiemstra, Maynard Meiring, Osagie A Eribo, Christian Otum, Ilana van Rensburg, Ayanda Shabangu, Bronwyn Smith, Firdows Noor, Gerhard Walzl, Kevin B Urdahl, Dave Lewinsohn, Stephanus T Malherbe, Nelita du Plessis

**Affiliations:** 1 Stellenbosch University, Cape Town, South Africa; 2 University of Washington School of Medicine, Washington, Seattle, USA; 3 Oregon Health and Science University and Portland Veterans Administration Medical Center, Portland, Portland, USA

**Keywords:** coinfection, SARS-CoV-2, COVID-19, Tuberculosis, Mtb, killing, peripheral blood mononuclear cells, immune response

## Abstract

**Background:**

There is a concern that SARS-CoV-2 infection may drive poor outcomes after *Mycobacterium tuberculosis* Mtb exposure and infection. We performed an *ex vivo* Mtb killing assay using peripheral blood mononuclear cells (PBMC) from three groups: healthy household contacts of people with active TB with and without serologic evidence of previous SARS-CoV-2 infection (COV+HHC and COV-HHC), and participants with active TB and previous SARS-CoV-2 (COV+TB+).

**Methods:**

Twenty participants per group from Cape Town, South Africa were classified according to SARS-CoV-2 anti-S and anti-N antibody tests. We infected PBMC from each participant at a MOI of 0.001 with Mtb strain H37Rv in a 4-day growth inhibition assay. Mycobacteria were quantified through inoculation into Bactec Mycobacteria Growth Indicator Tube (MGIT) liquid culture. PBMC from a subset of participants were infected in the presence of autologous time-matched serum and Mtb-uninfected control PBMCs were included.

**Results:**

There was no difference in the time to detection of Mtb or the normalised Mtb growth ratio (log10CFUsample – log10CFUcontrol) between groups in the standard protocol, or when infected cells from the COV+HHC and COV+TB+ (n=10 each) groups were cultured with autologous time-matched serum. The group with active TB demonstrated the best Mtb growth control. Extracellular Mtb measured by culturing the supernatants of the infected cell cultures also did not show any difference between groups. Five (14.3%) uninfected controls were culture positive.

**Conclusion:**

Our results show that previous SARS-CoV-2 does not affect the Mtb killing ability of circulating mononuclear immune cells *in vitro.* Previous SARS-CoV-2 is unlikely to affect the outcome of Mtb infection through this mechanism.

## Background

There is a high prevalence of severe acute respiratory syndrome coronavirus 2 (SARS-CoV-2) in populations which also have a high prevalence of tuberculosis (TB). ^1– 4^ Epidemiologic studies have shown that concomitant *Mycobacterium tuberculosis* (Mtb) infection worsens the prognosis of COVID-19. ^5– 7^ Moreover, there is concern that the temporary immunosuppressive effect associated with many viruses may lead to progression of subclinical or reactivation of latent mycobacterial infection. ^8, 9^ Coinfection may therefore have important implications for public health.

Murine studies seem to suggest that primary SARS-CoV-2 infection has little to no impact on the outcome of subsequent Mtb infection. ^10– 12^ Data in humans are very limited at present. *In vitro* and *ex vivo* studies have provided some evidence that primary infection with Mtb may not protect against SARS-CoV-2, but rather impair critical T cell responses to SARS-CoV-2. ^8, 13– 15^ Studies which indirectly examine the impact of primary infection with SARS-CoV-2 on the outcome of Mtb infection in humans often focus on cohorts with severe COVID-19 and these suggest that recent or concomitant SARS-CoV-2 may adversely affect the outcome of Mtb infection. ^8, 15– 19^

Monocyte dysfunction is one potential mechanism underlying this interaction. Mtb-influenza coinfection is known to worsen TB outcomes through alteration of the anti-mycobacterial activity of monocytes/macrophages in the lung, and influx of Mtb-susceptible myeloid cells that serve as a reservoir for Mtb. ^20– 22^ A dysregulated peripheral blood myeloid compartment is a feature of both COVID-19 and TB. ^23– 25^

*In vitro* growth inhibition of mycobacteria by peripheral blood mononuclear cells (PBMC), of which 15% - 20% are monocytes, is an objective measure of Mtb control by peripheral immune cells, and correlates with total pathology at necropsy, lung lesion count, and erythrocyte sedimentation rate after *in vivo* Mtb challenge in non-human primates. ^26, 27^ In human studies, a PBMC-based assay using Mycobacteria Growth Indicator Tube (MGIT) culture found that mycobacterial growth inhibition was better in people recently exposed to Mtb than in those with latent infection, and correlated with the presence of CD14 ^dim^ monocytes. ^28^ A whole blood growth inhibition assay using BCG-lux found no difference between Tuberculin skin test negative or positive household contacts (HHC) though this observation may depend on local TB prevalence. ^29, 30^ These authors also showed a correlation between Mtb growth inhibition and peripheral blood neutrophil counts in HHC, which was absent in healthy controls. ^29^ Lastly, altered proportions of intermediate and non-classical monocyte subsets correlated with differential mycobacterial control in whole blood from people with active TB disease compared to controls. ^31^

As most SARS-CoV-2 infections result in clinically mild disease, ^32^ we tested whether recent previous mild SARS-CoV-2 infection affects the ability of PBMC to inhibit Mtb growth, by adapting and implementing a mycobacterial growth inhibition assay (MGIA) previously optimised for BCG vaccine trials. ^8, 9^ We modified it for our in-house Mtb H37Rv strain, and compared the outcomes in healthy HHC (with known exposure to Mtb and therefore presumed recent Mtb infection) with evidence of previous SARS-CoV-2 infection, HHC without evidence of previous SARS-CoV-2 infection, and people with both active TB disease and previous SARS-CoV-2.

## Methods

### Sample population and recruitment

People with active TB disease and healthy HHC aged 18-65 years were recruited in the Western Cape, South Africa between July 2020 and September 2022, under two National Institutes of Health-funded projects, Cascade Immune Mechanisms of Protection against Mycobacterium tuberculosis (IMPAc-TB), ^33^ and Evaluation of New Diagnostics for Active and Incident TB (ENDx-TB).

Active TB was excluded in all HHC by symptom screen, chest radiograph, sputum smear microscopy, Bactec MGIT culture (Becton Dickinson, UK), and GeneXpert MTB/RIF Ultra (Xpert Ultra, Cepheid, Sunnyvale, USA). In HHC with previous COVID-19, subclinical TB was also ruled out with Positron Emission Tomography-Computed Tomography.

Active TB disease was defined as a person with positive sputum Xpert Ultra (>trace) or Bactec MGIT culture, and symptoms of active TB. Samples were collected before the initiation of TB treatment. People with drug-resistant TB were excluded. Strain typing was not performed.

Previous SARS-CoV-2 was identified using anti-nucleocapsid (N) antibody and anti-spike (S) receptor binding domain antibody assays (SARS-CoV-2 IgG and SARS-CoV-2 IgG II Quant on the Architect iSystem, Abbott Laboratories, Sligo, Ireland). People who only tested anti-S antibody positive were considered to have had SARS-CoV-2 if they had not been vaccinated with a SARS-CoV-2 anti-S antigen-based vaccine, as previously described. ^33^ A participant was classified as having had mild SARS-CoV-2 disease if they tested serology positive but had never experienced symptoms, or were symptomatic but did not require hospitalisation.

The parent studies were approved by the Stellenbosch University Health Research Ethics Committee (references N19/10/150 and N20/04/052), and all participants provided written informed consent.

### PBMC isolation and thawing

PBMC were isolated from heparinised blood samples using Ficoll density centrifugation and an optional red cell lysis step. Cells were counted, then resuspended in fetal bovine serum (FBS) with 10% dimethylsufoxide at a concentration of 10×10 ^6^ cells per vial (COV+HHC and COV+TB+ in one study) or 3-5×10 ^6^ cell per vial (COV-HHC in the other study), frozen overnight at -80 °C and transferred to liquid nitrogen for long term storage the next day. On the day of the MGIA, cryopreserved PBMC were gently thawed in a waterbath at 37 °C until a small part of the sample remained frozen, and added dropwise to warmed media (Roswell Park Memorial Institute [RPMI] with 20% FBS, 2 mM L-glutamine and sodium pyruvate). The cryovial was rinsed with fresh media, which was added to the rest of the sample and centrifuged at 300 g for 8 min. The supernatant was decanted and a second wash step performed, after which cells were rested for 2 h in a 37 °C incubator with 5% CO _2_, counted and resuspended at a concentration of 3×10 ^6^ cells per 300 μl in RPMI with 2 mM L-glutamine and 25 mM HEPES (4-(2-hydroxyethyl)-1-piperazineethanesulfonic acid). Serum was collected and stored separately at -80 °C.

### Preparation of mycobacterial stock

An in-house Mtb H37Rv strain (Primary strain: *Mycobacterium tuberculosis* subsp. *tuberculosis* ATCC ^®^ 27294™ (H37Rv - TMC 102), 12 passages) was grown in 7H9 liquid media [2.4 g Middlebrook 7H9 agar (BD #271310), 450 ml distilled water, 0.2% Glycerol (1ml Glycerol), 10% Oleic acid-Albumin-Dextrose-Catalase (OADC) enrichment (BD #212240), 0.05% Tween80] and cultured until mid-log phase, then homogenised and divided into 1 ml aliquots, and stored at −80 °C until required. For the MGIA, aliquots were thawed at room temperature immediately before inoculation, homogenised, and diluted in RPMI (with 2 mM L-glutamine and 25 mM HEPES) to a final concentration of 3000 colony-forming units (CFU) per 180 μl.

The stock standard curve was generated by triplicate plating and duplicate inoculation into Bactec MGIT tubes (supplemented with 800 μl premixed OADC growth enrichment and lyophilised polymyxin-B, amphotericin-B, nalidixic acid, trimethoprim, azilocillin (PANTA), supplied by Becton Dickinson, UK) of serial dilutions as previously described. ^26^ The time to detection (TTD) from the Bactec MGIT was plotted against log _10_CFU for each dilution and regression analysis used to obtain a regression line. Using the equation describing the line (Y = A*X+B -> X = (Y-B)/ A where A = slope and B = y-intercept, X = log10 CFU, Y = TTD), the log _10_CFU could be calculated by inserting the TTD for any given sample.

### Mycobacterial growth inhibition assay

The MGIAs were performed according to recommendations by Tanner *et al*, with modifications. ^26^ In the standard infection protocol, PBMC from each sample were infected with H37Rv at a multiplicity of infection (MOI) of 0.001 (3000 CFU of Mtb per 3×10 ^6^ PBMC, or an MOI of 0.007 for monocytes only) for 96 hours on a 48-well low-adherence plate (3×10 ^6^ cells/well) in RPMI supplemented with 2 mM L-glutamine, 10% heat-inactivated filtered pooled human serum (Merck Life Science (Pty) Ltd., Modderfontein, South Africa) and 25 mM HEPES at 37°C in 5% CO _2_. As only one aliquot was available per participant, in samples where fewer than 3×10 ^6^ PBMC were available post-thaw, all cells were used for the standard protocol with a proportionately adjusted inoculum volume.

For a subset of participants of the COV+HHC and COV+TB+ groups, the experiment was repeated with the participant’s autologous time-matched serum instead of pooled human serum. For a further subset of samples from all groups, PBMC were cultured for 96 hours in RPMI with 2 mM L-glutamine, 10% heat-inactivated filtered pooled human serum and 25 mM HEPES at 37 °C in 5% CO _2_, in the absence of Mtb.

Lastly, for each infection, Mtb inoculum controls were prepared as two labelled BACTEC MGIT tubes supplemented with 800 μl PANTA/OADC enrichment and 320 μl of extra supplemented Middlebrook 7H9, then inoculated with the same volume of the Mtb inoculum mix used to infect the PBMC.

After 96 hours incubation, PBMC in each well were mixed by pipetting and transferred to 2 ml screw cap tubes which were centrifuged at 12000 rpm for 10 min. The supernatant was carefully removed by pipetting, inoculated into a supplemented Bactec MGIT tube for a subset only. To capture any residual cells remaining in the well, 500 μl sterile tissue-culture grade water was added to each emptied well, incubated at room temperature for 10 min for cell lysis, and the contents added to the corresponding 2 ml screw cap tube containing the corresponding cell pellet. Tubes were pulse vortexed and the contents inoculated into a supplemented Bactec MGIT tube. All Bactec MGIT tubes were inverted to mix and then placed in the Bactec 960 machine (Becton Dickinson, UK). To account for potentially different bacterial inoculum used in each MGIA batch, the ‘Normalised Mtb growth ratio’ (log _10_CFU sample – log _10_CFU control) was calculated for each sample using the mean TTD from the two Mtb inoculum controls.

### Statistical analyses

*Sample size calculation and sample selection*

Selecting a 1-log change in the CFU/ml to be a biologically significant difference between two groups, and an expected variance (or standard deviation) of between 1.64 and 4.34 logCFU/ml, a power of 0.8 required a sample size of 13 participants in each of the groups. A group size of 20 yielded a power of >0.9.

*Analysis*

Statistical analysis was performed in R, Version 4.3.2. Continuous variables were expressed as medians with range, and categorical variables as number and percentage. For the main outcomes and clinical variables, means were compared between groups using one-way ANOVA. Bonferroni correction for multiple tests was applied. A multivariate linear model was generated for each outcome which included all the clinical variables: age, sex at birth, smoking status, illicit drug use and Haemoglobin concentration in blood (Hb) at the time of PBMC collection. Paired samples in the standard protocol and the autologous serum controls were analysed using a paired test on the medians (that is, the second quantile in a function comparing the marginal distributions of the two dependent groups). A p value <0.05 was considered significant.

## Results

### Sample population

The sample comprised 60 participants, 20 each in the HHC with serologic evidence of previous SARS-CoV-2 group (COV+HHC), the HHC without serologic evidence of previous SARS-CoV-2 group (COV-HHC), and the active TB disease with serologic evidence of previous SARS-CoV-2 (COV+TB +). The demographic and relevant clinical variables of the groups is shown in Table 1. Only three participants across all groups had any comorbidities, all of which were hypertension. All participants had either mild or asymptomatic COVID-19.

**Table 1.** Characteristics of the sample population.

	All (n=60)	COV-HHC (n=20)	COV+HHC (n=20)	COV+TB+ (n=20)
**Female**	27 (45)	11 (55)	11 (55)	5 (25)
**Age** (median, IQR)	29 (13)	29.5 (13)	29 (15)	29 (13)
**Hb** (g/dL) (median, IQR) ^a^	13.5 (1.58)	14.1 (1.72)	13.3 (2.00)	13.2 (1.62)
**Previous treated TB disease** ^a^	7 (11.7)	0	0	7 (35)
**Current tobacco smoking**	41 (68.3)	14 (70)	12 (60)	15 (75)
**Illicit substance use** ^a, b^	20 (33.3)	2 (10)	7 (35)	11 (55)

Numbers are n (%) unless otherwise indicated.

^a^ Between-group differences were significant at p<0.05. One-way ANOVA for differences in categorical variables; independent samples Kruskal-Wallis test for differences in continuous variables, with Bonferroni correction for multiple tests.

^b^ Illicit substances included smoking of cannabis, methamphetamine, or methaqualone. Hb, haemoglobin; IQR, interquartile range expressed as the difference between the 25 ^th^ and 75 ^th^ centiles.

### MGIA results

**Figure 1.**
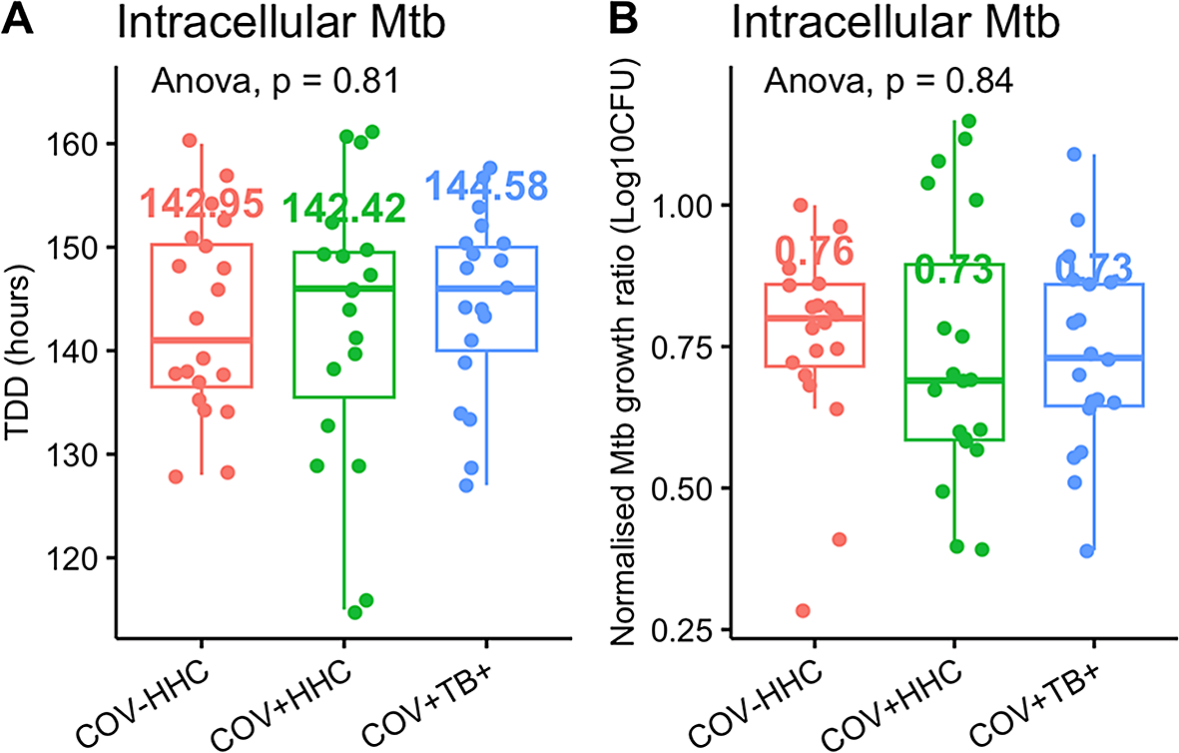
Previous SARS-CoV-2 does not affect *in vitro* Mtb growth inhibition by peripheral blood mononuclear cells. Panel A shows the time to detection of Mtb in the Bactec MGIT tubes (TTD) in the standard infection protocol, and panel B shows the normalised Mtb growth ratio (calculated as log _10_CFUsample – log _10_CFU control). A lower normalised Mtb growth ratio implies better Mtb growth inhibition.

There was no difference in Mtb growth inhibition between groups in the standard infection protocol, including when outliers were trimmed ( Figure 1).

**Figure 2.**
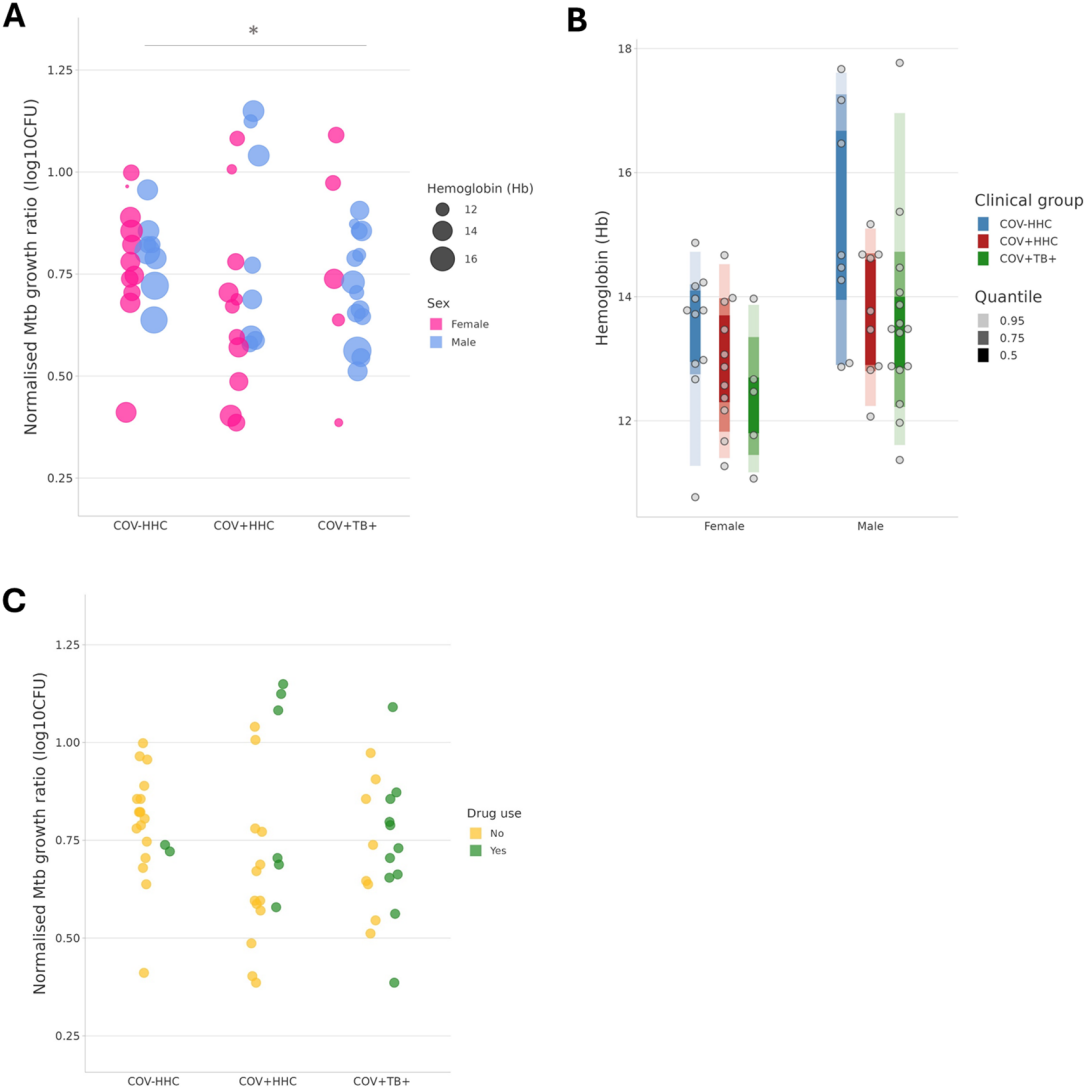
Peripheral blood mononuclear cells from people with current TB showed the best Mtb growth inhibition *in vitro* in a multivariate linear model. Panel A shows that the normalised Mtb growth ratio is different between groups when the two other variables which had an effect are considered: sex at birth, and participant Haemoglobin (Hb, in g/dL). * p<0.05. Panels B shows the relationship between sex at birth, clinical group and Hb levels in participants. Panel C illustrates the normalised Mtb growth ratios of samples stratified by illicit drug use. All four of the samples with the highest normalised Mtb growth ratios (poorest Mtb growth inhibition) were from people who were using illicit drugs. The proportion of drug users was higher in the COV+TB+ than the other groups.

When all covariates (age, sex at birth, smoking status, illicit drug use, and Hb) were considered in a linear model, the Mtb growth inhibition was significantly better in the COV+TB+ group than in the COV-HHC group (p=0.037) ( Figure 2A). The linear model also showed that Hb was independently associated with, and was negatively correlated with, the normalised Mtb growth ratios (p=0.024), which was explained by the effects of sex at birth and clinical group on the Hb when tested with two-way ANOVA on the means ( Figure 2B). Illicit drug use was not significant in the model overall, however all four of the samples with the worst Mtb growth inhibition were from people who were using illicit drugs ( Figure 2C).

**Figure 3.**
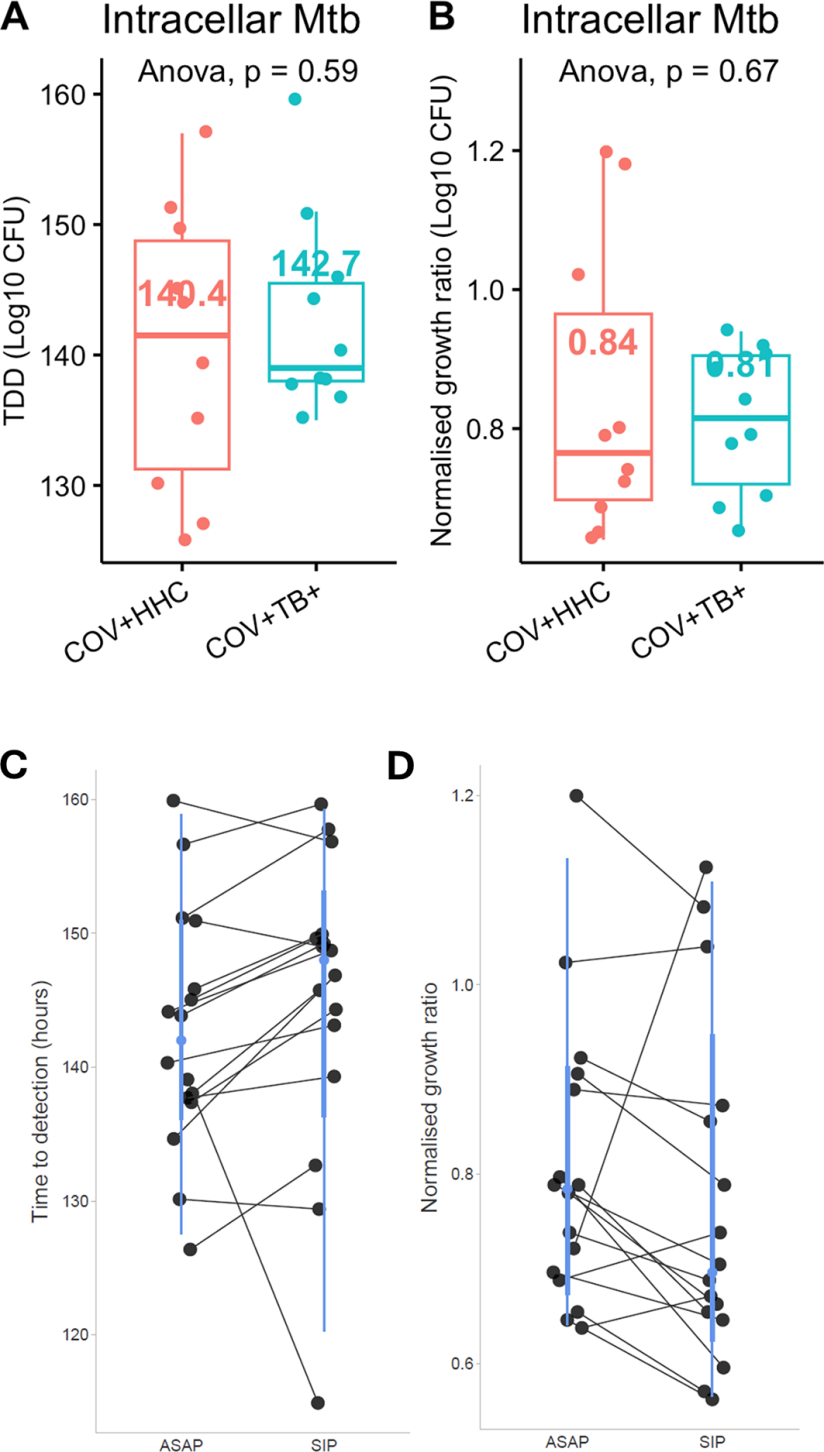
Incubating Mtb-infected peripheral blood mononuclear cells with autologous time-matched serum did not affect Mtb growth inhibition. Panel A shows the time to detection of Mtb, and Panel B shows the normalised Mtb growth ratio (log _10_CFUsample – log _10_CFUcontrol) of the samples incubated with autologous time-matched serum in the COV+HHC and the COV+TB+ groups. Panel C and D show the time to detection of Mtb and normalised Mtb growth ratios of the paired samples analysis between samples which had the standard infection protocol (SIP) and the autologous serum protocol (ASAP). P<0.05 for the time to detection of Mtb suggesting a longer TTD for the SIP group, but not significant for the normalised Mtb growth ratios.

In the autologous serum protocol, there was no significant difference in Mtb growth inhibition between PBMC from the COV+HHC (n=10) and COV+TB+ (n=10) groups ( Figure 3A and B). A comparison of paired samples between standard protocol infections and those with autologous serum (n=18) also found no significant difference in Mtb growth inhibition, although the TTD appeared higher in the standard protocol cultures ( Figure 3C and D).

As controls, uninfected PBMC were cultured from 6 of the COV-HHC group, 14 of the COV+HHC group, and 15 of the COV+TB+ group. Five (14.3%) of the 35 samples were positive for Mtb. Three were in the COV+HHC group (3/20 HHC total, 15.0 %) and two were in the COV+TB+ group (2/15, 13.3%). The median (IQR) TTD was 335 (153.5) hours, and median (IQR) mycobacteria was 15.85 (281.91) CFU/ml. Visual assessment found no suggestion of contamination in these Bactec MGIT tubes, but speciation was not performed. Subtracting this ‘background’ Mtb growth from the results of the standard protocol did not affect the results of the main analysis.

**Figure 4.**
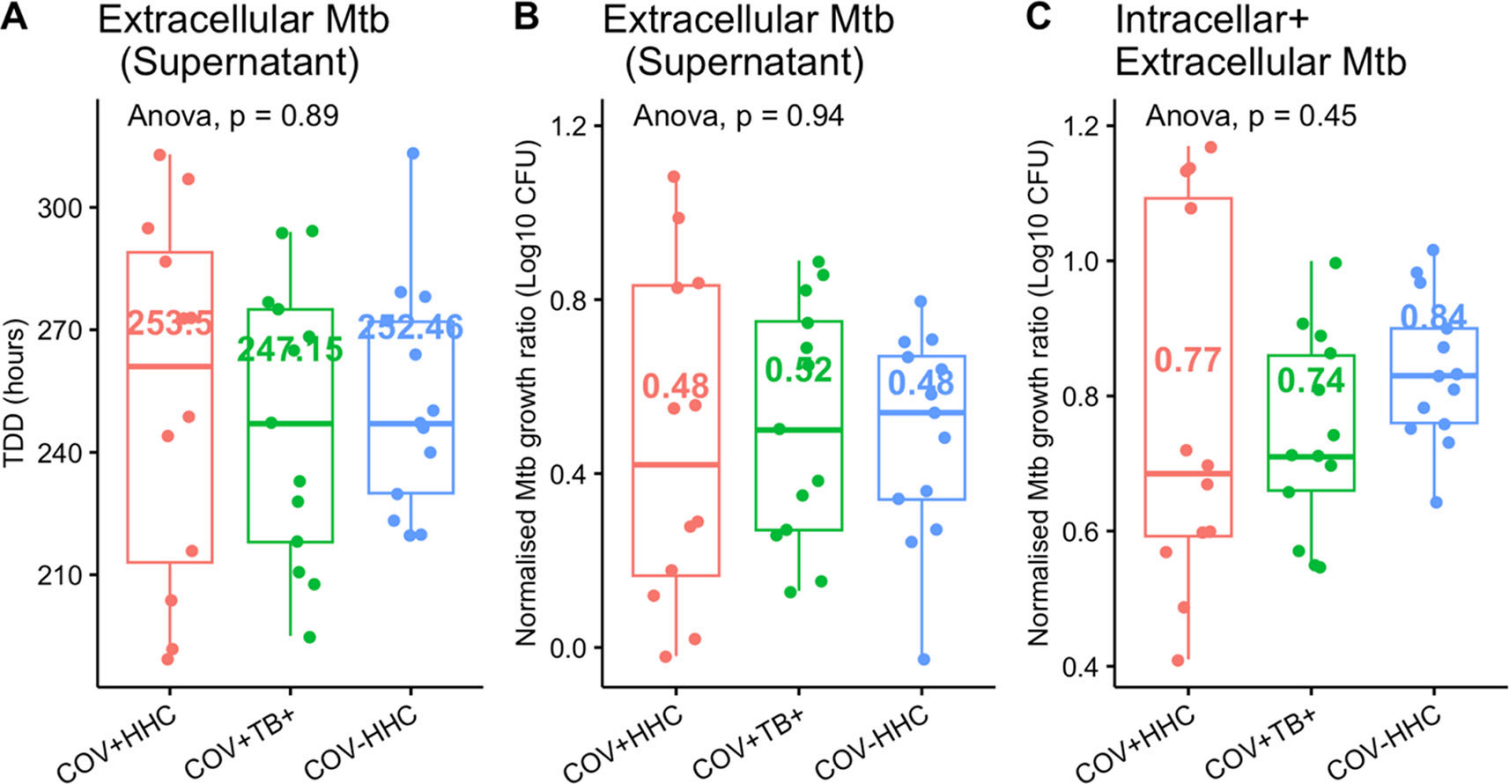
Extracellular mycobacteria did not differ between groups. Panel A and B show the time to detection of Mtb in the supernatants of the infected cells (n=35) and the corresponding normalised Mtb growth ratios (calculated as log _10_CFUsample/log _10_CFUcontrol to accommodate the negative numbers when low CFU counts were logged). Panel C shows the normalised Mtb growth ratios of the combined intracellular Mtb (from the pelleted cells) and the extracellular Mtb compared between groups.

The median (IQR) cell viability post-thaw was 93.4% (4.7%) with no significant differences between groups. Eleven of the 60 samples (18%) had fewer than 3×10 ^6^ available cells post thaw. Due to study-specific differences in PBMC storage concentrations, the COV-HHC group had significantly more samples with less than 3×10 ^6^ cells available. However, the results of the analysis did not change when adjusted for this covariate. Moreover, the infected cell culture supernatants were inoculated into separate Bactec MGIT tubes (n=38), to ascertain the proportion of bacteria which remained extracellular after 96 hours. The median (IQR) proportion of total Mtb that were extracellular after 96 hours infection was 1.97% (3.03%), with no significant differences between groups ( Figure 4). This implies that reduced cell numbers in culture did not affect the proportion of Mtb which were internalised.

The median inoculum control was 3.47 log _10_CFU, with a maximum difference of 0.43 log _10_CFU between batches, and no significant differences between groups.

## Discussion

Our experiments show that previous mild SARS-CoV-2 infection does not affect the *in vitro* Mtb H37Rv growth inhibition by PBMC, including when autologous serum is added to test the additional mycobactericidal impact of non-cellular blood components. A linear model which adjusted for the available clinical covariates found that PBMC from people with current TB disease and previous mild SARS-CoV-2 infection demonstrated better Mtb growth inhibition than the group without CoV infection and without active TB disease, in keeping with evidence from whole blood MGIAs, possibly due to priming of immune cells against Mtb. ^34^ These findings suggest that people in high SARS-CoV-2 prevalence areas, where most will manifest only mild or asymptomatic COVID-19, may not have impaired ability to eradicate new or existing Mtb infection.

There is strong epidemiologic evidence showing worse clinical outcomes – more severe disease and higher mortality – from concurrent Mtb/SARS-CoV-2 infection in humans, which suggested our hypothesis that SARS-CoV-2 impairs Mtb killing. Our results do not support such a conclusion for the long term effects of SARS-CoV-2 on Mtb infection. This may be because the outcome of Mtb infection is only affected by severe COVID-19, which is known to cause generalised T cell exhaustion, reduced circulating regulatory T cells, CD8 ^+^ T cells, natural killer cells, and CD4 ^+^ T cells, thereby possibly impairing the immune response to Mtb. ^35^ This is supported by Riou *et al*. who showed that the frequency of Mtb-specific CD4+ T cells is 5-fold lower in hospitalised patients with mild-moderate and severe acute COVID-19, than in a pre-pandemic cohort with latent TB infection. ^8^ It may be that our cohort of people with recent mild SARS-CoV-2 had no residual effects on their immune system which were significant enough to impair *in vitro* Mtb growth control.

A second possible explanation is that the observed increase in mortality from coinfection is attributable to non-immune factors that affect both COVID-19 and TB outcomes, such as nutritional status, delayed presentation, or pandemic-related limitations in health care service provision. This would be in keeping with evidence from murine studies. Rosas Mejia *et al*. showed that Mtb-infected mice were resistant to the pathological consequences of secondary SARS-CoV-2 infection, but that SARS-CoV-2 infection did not affect Mtb burdens in the lungs or distant organs. ^10^ Baker *et al*. also found no difference in TB burden between mice with prior SARS-CoV-2 and those without, and that Mtb/SARS-CoV-2 coinfection did not affect the proportions of pulmonary Mtb-specific CD4+ and CD8+ T cells, but did significantly reduce the proportion of SARS-CoV-2-specific CD4+ and CD8+ T cells. ^12^

Lastly, the higher mortality from coinfection reported in epidemiologic studies might be explained by the dysfunction of an element of the immune system, or at an anatomical site, or in a TB disease state which is not captured by our PBMC-based MGIA. In an analysis of transcriptomic signatures of PBMC and bronchoalveolar lavage fluid from people with SARS-CoV-2 infection and people with Mtb infection across all levels of severity, integrative single-cell-RNAseq analysis identified *FCN1*- and *SPP*-expressing macrophages enriched in both SARS-CoV-2 BALF and TB blood. ^13^ Moreover, human macrophages cultured in the inflammatory milieu of Mtb-infected macrophages showed increased susceptibility to SARS-CoV-2 infection *in vitro*, which correlated with the induction of several proinflammatory genes. ^13^ The interaction may therefore be macrophage-specific, or reliant on the lung microenvironment to manifest. A PBMC-based assay also excludes granuloctyes, which are an important part of both the Mtb and SARS-CoV-2 innate immune response. ^23, 36– 39^ Additionally, PBMC contains no serum, so it must be manually added to the culture wells. Autologous serum may be superior to pooled human serum, as the experiment then captures the additive effect of the serum-soluble components of the immune response. ^40, 41^ However, this is not always feasible. Our results on a subset of participants suggest that serum-derived factors did not contribute to Mtb control by peripheral immune cells. Lastly, our infection model does not capture the full spectrum of TB disease, which often lasts months to years, traversing various levels of symptomatology and severity, and potentially eliciting different immune responses than those which are elicited by a four-day *in vitro* infection.

An interesting finding of this experiment was the >14.0% of samples which grew very small numbers of Mtb from uninfected PBMC in the COV+ groups, suggesting circulation of viable Mtb inside cells at the time the specimens were collected. CD34 ^+^ haemopoietic stem cells (HSCs) are likely one peripheral immune cell type harbouring these Mtb bacilli. Mtb DNA has been detected in circulating CD34 ^+^ PBMC from asymptomatic TB contacts irrespective of latent infection status, both in people with and without HIV. ^42^ In mice, Mtb from CD34 ^+^ long-term repopulating pluripotent HSCs begin to replicate and cause disease when transferred from donor mice without disease into immune-deficient mice. ^43^ CD34+ HSCs express the cell surface receptor used by SARS-CoV-2, rendering them potentially susceptible to infection. ^44^ Exposure to the SARS-CoV-2 S protein causes phenotypical and functional changes in CD34+ HSCs *ex vivo.* ^44^ This suggests the possibility that prior SARS-CoV-2 infection results in the circulation of dysfunctional CD34+ HSCs, which may also be more susceptible to Mtb. Unfortunately, these positive cultures were not sequenced, so we were unable to definitively exclude contamination from the lab strain.

Our study may be limited by our sample collection spanning all four major waves of the pandemic (including wild type, beta, delta and omicron variants), introducing a possible confounder. Another limitation is that participants were classified into the SARS-CoV-2 groups based on serology only, and as most of them were asymptomatic, we were unable to reliably quantify the time since infection. According to the antibody kinetics of SARS-CoV-2 infection, infection could have occurred anywhere between 4 days and 6 months before the date of testing. This might explain the variability observed between the samples in the COV+ groups. Importantly, our findings do not address or exclude an interaction between Mtb infection and severe COVID-19.

Future work in the field of Mtb/SARS-CoV-2 coinfection should explore the immune responses of lung immune cells, including the effect of SARS-CoV-2 on cytokine production and the gene transcription in response to new Mtb exposure. An important target group is people with subclinical TB, to ascertain whether SARS-CoV-2 might be one of the factors determining their disease course.

In conclusion, we have provided the first direct evidence that the most prevalent form of COVID-19, mild SARS-CoV-2 infection, does not affect the efficacy of Mtb killing by circulating mononuclear immune cells.

## Meeting presentations

This work was presented at the European Respiratory Society Congress in Vienna, Austria on 9 September 2024.

## Patient consent statement

This research was conducted in accordance with the Declaration of Helsinki. All participants provided written informed consent. These studies were approved by the Stellenbosch University Health Research Ethics Committee, reference numbers: N19/10/150 and N20/04/052, and all participants provided written informed consent.

## Corresponding author contact information

Associate Professor Jane A Shaw

janeshaw@sun.ac.za

Room 2020 BMRI South, Francie Van Zijl Drive, Tygerberg, 7505

Stellenbosch University Tygerberg Medical Campus|South Africa

+27 21 938 9953|+27 76 530 6682

## Author contributions

JAS, NdP, GW, KU, STM conceived and designed the study; AH, AS, BS, IvR, FN, JAS collected and contributed to the data and samples; JAS and CP performed the experiments with assistance from CO and OE; MM and JAS performed the statistical analyses with assistance from OE; KU, IvR, BS, FN and DL contributed toward project management and execution, JAS wrote the paper, which was critically reviewed, edited and approved in the final draft by all authors.

## Data Availability

Figshare: Mycobacterium tuberculosis growth inhibition by peripheral blood mononuclear cells from household contacts is not affected by previous SARS-CoV-2 infection, DOI: https://doi.org/10.6084/m9.figshare.28007885.v1. ^45^ The project contains the following underlying data:
•
MGIA_results_11Dec2024 Data are available under the terms of the Creative Commons Attribution 4.0 International license (CC-BY 4.0).
